# Prof. Ebstein’s Diet for the Corpulent

**Published:** 1884-06

**Authors:** 


### PROF. EBSTEIN'S DIET FOR THE CORPULENT.

The annexed bill of fare is that proposed by Professor Ebstein for an average case of corpulency, the invalid being supposed to be forty-one years of age and having suffered from increasing stoutness for twenty-five years. The disease is supposed to be contracted by insufficient bodily exercise, a diet consisting of such things as are hurtful, among which are named all sweet dishes and those containing much albumen and those devoid of a sufficient quantity of fat.

Breakfast.—A large cup of black tea without milk or sugar, fifty grams of white bread or toasted brown bread, with plenty of butter.

Dinner.—Soup (frequently and with bone marrow), one hundred and twenty to one hundred and eighty grams meat, boiled or roasted, with fat gravy—fat meat being preferable—a small quantity of vegetables, particularly leguminous, but also all kinds of cabbage. Turnips are excluded because of the sugar contained in them; potatoes are altogether excluded. After dinner some fresh fruit, when in season, as dessert; a salad or baked fruit without sugar; two or three glasses of light wine. After dinner a large cup of black tea, without milk or sugar.

Supper.—In winter regularly, in summer occasionally, a large cup of black tea, without milk and sugar, an egg or some fat roast meat, or both, sometimes fat ham, smoked or fresh fish, about thirty grains of white bread, with plenty of butter, and occasionally a small quantity of cheese and some fresh fruit. —*Ex.*

